# Optimising the Production Process of Bacterial Nanocellulose: Impact on Growth and Bioactive Compounds

**DOI:** 10.17113/ftb.61.04.23.8182

**Published:** 2023-12

**Authors:** Nicole Folmann Lima, Giselle Maria Maciel, Isabela de Andrade Arruda Fernandes, Charles Windson Isidoro Haminiuk

**Affiliations:** 1 Postgraduate Program in Environmental Science and Technology, Federal University of Technology – Paraná, 5000 Deputado Heitor Alencar Furtado Street, 81280-340, Curitiba, PR, Brazil; 2 Biotechnology Laboratory, Department of Chemistry and Biology, Federal University of Technology – Paraná, 5000 Deputado Heitor Alencar Furtado Street, 81280-340, Curitiba, PR, Brazil; 3 Postgraduate Program in Food Engineering, Federal University of Paraná, 100 Coronel Francisco Heráclito dos Santos Avenue, 81530-000, Curitiba, PR, Brazil

**Keywords:** bacterial cellulose, fermentation optimisation, phenolic content, antioxidant activity, kombucha

## Abstract

**Research background:**

Research into bacterial cellulose production has been growing rapidly in recent years, as it has a potential use in various applications, such as in the medical and food industries. Previous studies have focused on optimising the production process through various methods, such as using different carbon sources and manipulating environmental conditions. However, further research is still needed to optimise the production process and understand the underlying mechanisms of bacterial cellulose synthesis.

**Experimental approach:**

We used Plackett-Burman and Box-Behnken experimental designs to analyse the effect of different factors on bacterial cellulose production. The fermentation kinetics of the optimised medium was analysed, and the produced cellulose was characterised. This approach was used because it allows the identification of significant factors influencing bacterial cellulose growth, the optimisation of the culture medium and the characterisation of the produced cellulose.

**Results and conclusions:**

The results showed that higher sucrose concentrations, higher kombucha volume fractions and a smaller size of the symbiotic culture of bacteria and yeast were the most important factors for the improvement of bacterial cellulose production, while the other factors had no relevant influence. The optimised medium showed an increase in the concentrations of total phenolic compounds and total flavonoids as well as significant antioxidant activity. The produced pure bacterial cellulose had a high water absorption capacity as well as high crystallinity and thermal stability.

**Novelty and scientific contribution:**

The study makes an important scientific contribution by optimising the culture medium to produce bacterial cellulose more productively and efficiently. The optimised medium can be used for the production of a kombucha-like beverage with a high content of bioactive compounds and for the production of bacterial cellulose with high crystallinity and thermal stability. Additionally, the study highlights the potential of bacterial cellulose as a highly water-absorbent material with applications in areas such as packaging and biomedical engineering.

## INTRODUCTION

Kombucha is a fermented drink that originates from northeast China. It is usually made by fermenting *Camellia sinensis* tea, with the most commonly used varieties being black and green tea. The process involves the fermentation of a symbiotic culture of bacteria and yeast (SCOBY) in a nutritious medium for approx. 7 to 10 days (*1*). During fermentation, the SCOBY consumes the sugar in the tea and produces a range of organic acids and enzymes, giving the kombucha its tangy and slightly sweet taste. As the fermentation progresses, a new layer of SCOBY forms on the surface of the liquid and can be used to start a new batch of kombucha (*2*, *3*). The process leads to the production of organic acids that increase their concentration in the broth, resulting in a decrease in its pH value. This pH drop causes a colour change in the broth, which is attributed to modifications in the phenolic compounds (*4*). Enzymatic activity on polyphenols is also believed to contribute to this colour change (*5*).

Due to the presence of various strains of bacteria and yeast, the fermentation of kombucha results in the production of ethanol and acetic, lactic, gluconic and glucuronic acids, as well as a large amount of phenolic compounds (*5*, *6*). The microorganisms associated with kombucha production are called chemoheterotrophs, as they derive energy and carbon from the decomposition of organic matter (*7*). Sucrose is one of the primary sources of carbon used in the kombucha fermentation; however, it is not completely utilised during the process (*8*). Green tea is the most commonly used for kombucha fermentation due to its better stimulant effect than other teas with the same parameters, resulting in a shorter production time (*9*).

The SCOBY consists mainly of cellulose, which is produced by acetic acid bacteria and has a high water retention capacity, crystallinity and thermal stability (*10*). Additionally, it has properties such as biodegradability, purity and biocompatibility, which opens up possibilities for its application in various fields (*11*). Although it is chemically comparable to plant cellulose, bacterial cellulose (BC) does not contain by-products such as lignin, pectin, hemicellulose or other components of lignocellulosic materials, making it a chemically pure form of cellulose (*12*). This makes it a highly versatile material that finds applications in multiple industries, including the food, biomedicine, pharmaceuticals, cosmetics and even bioengineering (*13*). In addition, a major challenge is making BC more attractive to the market since its current production is characterised by low efficiency, long production time and high costs (*14*).

As a result, several studies are being conducted on the use of industrial waste as an alternative substrate for BC production. These waste materials may contain nutrient sources that can be used as an energy source by bacteria, thus reducing production costs. Furthermore, the use of industrial waste for BC production can help address waste management problems with less impact on the environmental than conventional raw materials (*15*).

Coffee is one of the most consumed beverages in the world, and its worldwide consumption is expected to reach the equivalent of 10.2 billion kg in 2023 (*16*). Coffee grounds are rich in caffeine, amino acids, phenolic compounds, minerals and polysaccharides, making them a promising source for the production of BC. Furthermore, the use of coffee grounds as an alternative substrate can reduce the volume of waste generated by the coffee industry, thus contributing to waste management and environmental protection (*17*).

In this context, this study was conducted to optimise the production of bacterial cellulose through experimental designs. The justification for this work is the importance of optimising bacterial cellulose production to make it more efficient and sustainable, thus contributing to the reduction of costs and environmental impacts. In addition, bacterial cellulose is a versatile material with various applications in the industry, so optimising its production is a critical strategy to promote sustainable development. To achieve this objective, the Plackett-Burman design was used to identify the significant variables for bacterial cellulose growth, followed by the Box-Behnken design to determine the optimal culture medium.

## MATERIALS AND METHODS

### Microorganisms and raw materials

The starter culture (SCOBY) was obtained from a local source in the Curitiba region of Paraná, Brazil, and was maintained in a standard medium (70 g/L sucrose and 3 g/L green tea) in a bacteriological incubator at (28±2) °C. Sucrose p.a. grade (Neon, Suzano, Brazil) was used. The green tea and vitamin complex were purchased commercially from a natural products store in Curitiba, Paraná. The coffee grounds were kindly obtained from the Research and Development department of Café Iguaçu company in Cornélio Procópio, Paraná, Brazil.

### Culture medium selection

For the determination of factors that affect BC production, the Plackett-Burman (*18*) design was used, where seven independent variables were studied at higher (+1) and lower (-1) levels of 8 experiments, including nutrients such as sucrose, green tea, coffee grounds, vitamin complex and kombucha, as well as physical factors such as SCOBY size and incubation time. Based on the Plackett-Burman results, the most relevant variables were analysed. The Box-Behnken design (*19*) was then used to determine the optimal culture medium with three independent variables at the levels -1, 0 and +1 using 15 experiments. The parameter used to determine the best medium for BC growth for both designs was the daily production of BC (in g/(day∙L)), calculated according to the following equation:

BC production=(*m*_b_/*t*_c_∙*V*_c_) /1/

where *m*_b_ is the dry mass of BC (g), *t*_c_ is the cultivation time (day) and *V*_c_ is the culture medium volume (L).

### Production and treatment of BC

For the experiments, autoclaved distilled water was used to prepare 150 mL of culture medium and incubated in a bacteriological incubator (Vulcan, São Paulo, Brazil) at (28±2) °C. After determining the optimal culture medium, the membranes were purified with a 0.1 M NaOH solution at 80 °C for 2 h and washed with distilled water. To determine its production, the purified BC was dried at 50 °C for 48 h until it reached a constant mass (*20*).

### Fermentation kinetics

The kinetics of BC fermentation was evaluated for 7 days using the optimised culture medium formulation, which was determined to be the optimal BC production incubation time. For the fermentation kinetic evaluation, beakers containing 40 mL of culture medium were placed in a bacteriological incubator (Vulcan) at (28±2) °C and samples were collected in triplicate every 24 h. BC was collected daily and used to calculate the medium productivity. The fermented medium was then used for the analyses of pH, total phenolics, total flavonoids, antioxidant activity, reducing sugars and total proteins.

### pH and total acidity

The pH was measured for fermentation kinetics every 24 h, from day 1 to day 7 of fermentation. A benchtop pH meter (PG1800; Gehaka, São Paulo, Brazil) was used for the measurements. Total acidity was determined by gravimetric titration with previously standardised NaOH 0.1 mol/L until the endpoint was reached with the phenolphthalein indicator. The concentration of acetic acid was calculated using the following equation:

*c*_acetic acid_ ∙*V*_acetic acid_=*c*_NaOH_ ∙*V*_NaOH_ /2/

where *c* is the concentration (mol/L) and *V* is the volume (L).

### Colourimetric analysis

#### Total phenolic content

The total phenolic content (TPC) was determined using the Folin-Ciocalteu colourimetric method (*21*). Absorbance was measured at 765 nm using a spectrophotometer (UV-M51; BEL Engineering, Monza, Italy). Calculations were based on a gallic acid calibration curve (Sigma-Aldrich, Merck, São Paulo, SP, Brazil) and the TPC concentrations in the samples were expressed in mg gallic acid equivalents (GAE) per L.

#### Total flavonoids

The total flavonoids (TF) were determined using the aluminium chloride colourimetric method (*22*). Absorbance was measured at 510 nm using a spectrophotometer (UV-M51; BEL Engineering, Monza, Italy). The values were calculated based on a calibration curve obtained with the catechin standard (Sigma-Aldrich, Merck, St. Louis, MO, USA) and the results were reported in mg catechin equivalents (CE) per L.

#### Antioxidant activity

The antioxidant activity (AA) was determined using the 2,2-diphenyl-1-picrylhydrazyl (DPPH) (*23*) and 2,2'-azino-bis(3-ethylbenzothiazoline-6-sulfonic acid) (ABTS) (*24*) methods. The absorbance of DPPH was measured at 517 nm and ABTS at 734 nm using a spectrophotometer (UV-M51; BEL Engineering). The values were calculated based on calibration curves obtained with 6-hydroxy-2,5,7,8-tetramethylchroman-2-carboxylic acid (Trolox) (Acros Organics, Geel, Belgium) and expressed in μM.

#### Reducing sugars

Reducing sugars (RS) were determined using the 3,5-dinitrosalicylic acid (DNS) method (*25*). The absorbance was measured at 540 nm using a spectrophotometer (UV-M51; BEL Engineering). The calculations were based on a calibration curve obtained with glucose (Neon) and expressed in g/L.

#### Total protein

The total proteins (TP) were determined using the Bradford method (*26*). The absorbance was measured at 595 nm using a spectrophotometer (UV-M51; BEL Engineering). The calculations were based on a calibration curve obtained with bovine serum albumin (Sigma-Aldrich, Merck, St. Louis). Results were expressed in g/L.

### Water absorption capacity

The water absorption capacity (WAC) was determined by immersing the dry membranes in deionised water and storing them until equilibrium was reached (approx. 48 h) in an incubator (Vulcan) at (35±2) °C. The membranes were then removed from the water and excess water was removed from the surface. The mass of the hydrated membranes was measured using an analytical balance (Tecnal, São Paulo, Brazil) and the process was repeated until equilibrium was reached. WAC was calculated according to the following equation (*27*):

WAC=(*m*_h_-*m*_d_/*m*_d_)∙100 /3/

where *m*_h_ is the mass of the hydrated membrane (g) and *m*_d_ is the mass of the dried membrane (g).

### Crystallinity

The BC samples were analysed using a X-ray diffractometer (XRD-7000; Shimadzu, Kyoto, Japan) at the Multi-User Center for Material Characterisation (CMCM) of the Federal University of Technology, Paraná, Brazil. The equipment was operated at 30 kV and 30 mA with a scan rate of 2°/min and angles ranging from 5 to 100° (2*θ*).

### Identification of microstructures

The microstructures were identified using scanning electron microscopy (SEM) with a scanning electron microscope (EVO MA 15; Zeiss, Oberkochen, Germany) at the CMCM of the Federal University of Technology, Paraná, Brazil. For image preparation, the BC was coated with 90 nm of gold and the equipment was operated at 20 kV and magnified 500-5000 times until the membrane reached the maximum heat resistance of the equipment.

### Thermal stability

The thermal stability of the BC was determined by thermogravimetric analysis (TGA) and derivative thermogravimetric analysis (DTG) using the simultaneous TGA/DSC equipment (SDT Q600; TA Instruments, New Castle, DE, USA). Approximately 4−8 mg purified dry bacterial cellulose samples were used in a hermetically sealed aluminium crucible under a heating rate of 10 °C/min up to 600 °C using a nitrogen atmosphere with a flow rate of 50 mL/min.

### Statistical analysis

All analyses were performed in triplicate. The data were presented as mean value±standard deviation. Statistical analysis was carried out by analysis of variance (ANOVA), followed by Tukey's test to determine significant differences between samples with a 95 % confidence interval (p≤0.05), which was represented by superscript letters on the standard deviation values. Statistica 8.0 software (*28*) was used for the Plackett-Burman and Box-Behnken experimental designs and their respective analyses, as well as for all colourimetric, pH and total acidity analyses. OriginPro 2023 software (*29*) was applied for the determination of BC crystallinity and thermal stability analyses.

## RESULTS AND DISCUSSION

### Influence of culture medium on BC production

For the Plackett-Burman experimental design with eight replicates, each column represents an independent variable, and each row represents an experiment. The levels +1 and -1 represent the higher and lower levels of the analysed independent variables. The results of the Plackett-Burman design of the effects of seven culture variables on the production of BC and dry membrane mass are summarised in Table 1.

**Table 1:** Plackett-Burman design for seven variables and results for bacterial cellulose (BC) dry mass and daily production

Experiment	X_1_	X_2_	X_3_	X_4_	X_5_	X_6_	X_7_	*m*(BC)/g	BC production/(g/(day∙L))
1	6	3	500	70	5	10	10	(2.3±0.2)^ab^	(0.23±0.02)^cd^
2	6	3	1000	70	2.5	7	20	(1.94±0.00)^a^	(0.28±0.00)^de^
3	6	5	500	50	5	7	20	(1.48±0.09)^a^	(0.21±0.01)^bc^
4	6	5	1000	50	2.5	10	10	(1.00±0.02)^a^	(0.10±0.00)^a^
5	12	3	500	50	2.5	10	20	(1.8±0.4)^a^	(0.18±0.04)^a^
6	12	3	1000	50	5	7	10	(0.8±0.1)^a^	(0.14±0.00)^ab^
7	12	5	500	70	2.5	7	10	(1.33±0.63)^a^	(0.20±0.07)^a^
8	12	5	1000	70	5	10	20	(3.21±0.03)^a^	(0.32±0.00)^e^

X_1_=coffee grounds (g/L), X_2_=green tea (g/L), X_3_=vitamin complex (mg/L), X_4_=sucrose (g/L), X_5_=symbiotic culture of bacteria and yeast (SCOBY) (*m*/*V*), X_6_=incubation time (day), X_7_=kombucha (*V*/*V*)

**Fig. 1:**
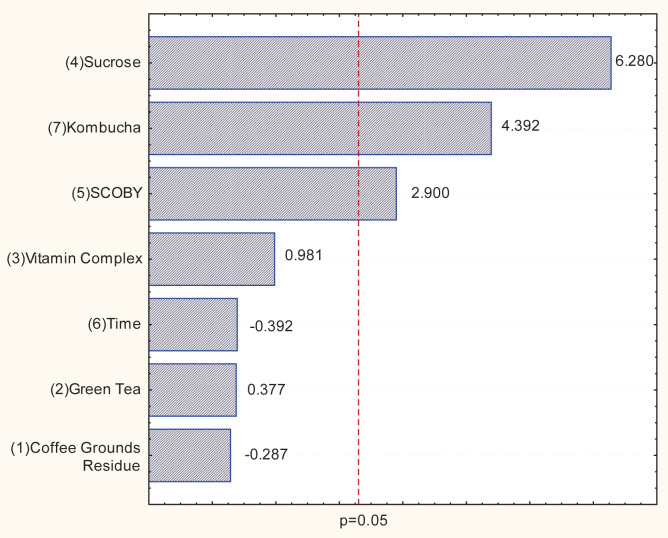
Pareto chart for the Plackett-Burman design. SCOBY=symbiotic culture of bacteria and yeast

The Plackett-Burman design was used to determine which variables significantly affect BC production. It can be seen that within the studied conditions, the best combination of nutrients and physical parameters was achieved in experiment 8, in which BC production ((3.21±0.03)/(g/(day∙L)) and dry membrane mass ((0.32±0.00) g) were the highest. In the Pareto chart (Fig. 1), it is possible to identify the variables that significantly affected BC production.

It was found that the concentrations of sucrose, kombucha volume fraction and SCOBY (*m*/*V*) had a significant positive effect on BC production at a confidence level of p<0.05. However, the concentrations of vitamin complex, green tea, coffee grounds and incubation time did not have significant effects. These results indicate that these factors may not be substantial for SCOBY production or may require further investigation to fully understand their effect on the production process. It is important to note that these results are specific to the current study and may vary depending on the experimental conditions used. Therefore, it is crucial to perform appropriate statistical analyses to evaluate the effect of the different factors and optimise the culture medium according to the specific requirements of each production process. A possible explanation for the fact that only some factors are significant could be the synergistic effect. The combination of the three relevant factors produces a more significant positive effect than if each factor were tested individually. This type of synergistic effect is common in complex biological processes.

As evident from the results, the optimal composition of the medium requires higher levels of sucrose, kombucha and SCOBY. Therefore, only the variables that showed significance according to the Plackett-Burman design were selected for the Box-Behnken experimental design. The Box-Behnken design was carried out with fifteen replicates, where each column represented an independent variable and each row represented an experiment. The levels +1, 0 and -1 represent the levels of the independent variables under study. The results of the Box-Behnken design on the effects of the three cultivation variables on BC production and membrane dry mass are summarised in Table 2.

**Table 2:** Box-Behnken design for three variables and results for bacterial cellulose (BC) dry mass and daily production

Experiment	X_1_	X_2_	X_3_	*m*(BC)/g	BC production/(g/(day∙L))
1	70	20	7.5	(2.8±0.1)^a^	(0.40±0.02)^a^
2	110	20	7.5	(3.44±0.04)^ab^	(0.49±0.01)^ab^
3	70	40	7.5	(3.4±0.1)^ab^	(0.49±0.02)^ab^
4	90	40	7.5	(5.3±0.2)^e^	(0.76±0.02)^e^
5	70	30	5	(3.5±0.1)^b^	(0.50±0.02)^b^
6	110	30	5	(4.7±0.1)^de^	(0.67±0.02)^de^
7	70	30	10	(3.18±0.07)^ab^	(0.45±0.01)^ab^
8	110	30	10	(4.4±0.1)^d^	(0.62±0.02)^d^
9	90	20	5	(4.55±0.08)^d^	(0.65±0.01)^d^
10	90	40	5	(6.4±0.3)^f^	(0.92±0.05)^f^
11	90	20	10	(4.3±0.2)^cd^	(0.61±0.04)^cd^
12	90	40	10	(4.6±0.2)^d^	(0.66±0.03)^d^
13	90	30	7.5	(3.7±0.2)^bc^	(0.53±0.02)^bc^
14	90	30	7.5	(3.40±0.09)^ab^	(0.49±0.01)^ab^
15	90	30	7.5	(3.2±0.2)^ab^	(0.46±0.03)^ab^

X_1_=sucrose (g/L), X_2_=kombucha (*V*/*V*), X_3_=symbiotic culture of bacteria and yeast (SCOBY) (*m*/*V*)

The Box-Behnken design was used to determine the optimal culture medium for bacterial cellulose production. To obtain the best optimisation conditions, the desirability function of the Statistica 8.0 program (*28*) was used to determine the optimal values for the studied parameters. The individual desirability of each variable was analysed to show the effect of each variable on the production of BC. The results showed that higher content of sucrose (+1), kombucha (+1) and lower content of SCOBY (-1) were the most significant factors in the optimal culture medium for bacterial cellulose production, resulting in a global desirability of 0.87.

Therefore, based on the ideal conditions for BC production, it was possible to obtain the optimised composition of the culture medium consisting of *γ*(sucrose)=110 g/L, *φ*(standard kombucha)=40 %, SCOBY 5 % (*m*/*V*) and *γ*(green tea)=3 g/L. These results can be used to optimise the BC production process and improve its efficiency on a large scale.

### Fermentation kinetics

The kinetic evaluation of the culture medium was used to determine the processes that occurred during kombucha fermentation and bacterial cellulose production. Table 3 summarises the results of TPC, TF and AA for the 7-day kinetics of the optimal culture medium. Table 4 shows the data obtained for the values of pH, total acidity, RS, TP, BC production and mass of dry BC.

**Table 3:** Total phenolics (TPC), total flavonoids (TF) and antioxidant activity (AA) of the culture medium during fermentation

Day	*γ*/(mg/L)		*c*(AA as Trolox)/µM	
TPC (as GAE)	TF (as CE)
		DPPH	ABTS
0	(372±13)^a^	(41.7±0.7)**^a^**	(945±6)^a^	(2346.75±0.02)^a^
1	(388±16)^a^	(41.2±0.8)^a^	(943±8)^ac^	(2451.00±0.08)^a^
2	(417±22)^ab^	(40.6±0.8)^a^	(926±7)^abc^	(2460.00±0.00)^a^
3	(474±15)^bc^	(40.42±0.09)^a^	(924±5)^abc^	(2306.25±0.00)^a^
4	(500.5±0.9)^c^	(40.2±0.4)^a^	(919±1)^bc^	(2227.25±0.00)^a^
5	(560±22)^d^	(41.15±0.08)^a^	(914±8)^bc^	(2350.75±0.01)^a^
6	(586±12)^d^	(47.0±0.7)^b^	(937±8)^abc^	(2592.50±0.03)^a^
7	(669±5)^e^	(51.1±0.1)^b^	(944±2)^a^	(2789.00±0.01)^a^

DPPH=2,2-diphenyl-1-picrylhydrazyl, ABTS=2,2'-azino-bis(3-ethylbenzothiazoline-6-sulfonic acid), GAE=gallic acid equivalents, CE=catechin equivalents

**Table 4:** pH, total acidity (TA), reducing sugars (RS), total proteins (TP), bacterial cellulose (BC) dry mass and daily production during fermentation

*t*/day	pH	*γ*(TA as acetic acid)/(g/100 mL)	*γ*(RS as glucose)/(g/L)	*γ*(TP as BSA)/(g/L)	*m*(BC)/g	BC production/(g/(day∙L))
0	(2.69±0.00)^c^	(0.40±0.00)^c^	(24.8±0.4)^a^	(0.59±0.00)^b^	(0.00±0.00)^a^	(0.00±0.00)^b^
1	(2.67±0.01)^c^	(0.6±0.02)^a^	(27.43±0.06)^b^	(0.61±0.00)^d^	(0.00±0.00) ^a^	(0.00±0.00)^b^
2	(2.59±0.01)^e^	(0.64±0.04)^a^	(30.07±0.08)^c^	(0.52±0.00)^a^	(4.58±0.09)^b^	(2.29±0.05)^d^
3	(2.55±0.01)^d^	(0.69±0.02)^ab^	(35.0±1.0)^d^	(0.53±0.00)^a^	(5.1±0.2)^bc^	(1.71±0.08)^c^
4	(2.49±0.01)^b^	(0.73±0.00)^ab^	(45.8±0.7)^e^	(0.53±0.00)^a^	(5.7±0.5)^c^	(1.4±0.1)^a^
5	(2.45±0.00)^b^	(0.77±0.04)^b^	(58.9±0.4)^f^	(0.56±0.00)^b^	(7.41±0.07)^e^	(1.48±0.01)^ac^
6	(2.41±0.01)^a^	(0.95±0.04)^d^	(64.4±0.5)^g^	(0.63±0.00)^e^	(8.8±0.4)^d^	(1.48±0.07)^a^
7	(2.38±0.00)^a^	(1.09±0.02)^e^	(77.0±0.4)^h^	(0.70±0.00)^f^	(9.8±0.2)^d^	(1.40±0.02)^a^

BSA=bovine serum albumin

#### Concentrations of TPC, TF and AA in kombucha

The fermentation of kombucha is known to positively affect the concentration of phenolic compounds in green tea, as these compounds are more stable in acidic than in alkaline solutions. Thus, the fermentation of kombucha can increase the concentration of phenolic compounds in tea (*5*). The findings indicate a significant growth in kombucha production using the optimal culture during fermentation. Specifically, the concentration of phenolic compounds in the fermented tea increased by an impressive 80 % within 7 days.

Chakravorty *et al.* (*6*) demonstrated that black tea fermentation resulted in a 54 % increase in total phenolic compound concentration after 21 days. On the other hand, Özdemir and Çon (*30*) observed an increase of about 20 % in phenolic compounds in green tea kombucha and 10 % in black tea kombucha. Gaggìa *et al.* (*31*) obtained a maximum increase of approx. 35 % on the 7th day of green tea fermentation. It is important to note that several factors can affect the variation of phenolic compounds in the medium, such as fermentation time, origin and type of tea used, as well as the composition of the starter culture (*32*). However, the optimal culture medium used in the current study showed a higher capacity for phenolic compound production than in other studies.

At the beginning of fermentation, bacteria and yeasts have greater metabolic activity, which can lead to the degradation of some flavonoids. However, as fermentation progresses, the metabolites produced by bacteria and yeasts begin to affect the activity of the enzymes, resulting in a better stability of flavonoids in the medium. In addition, some metabolites produced during fermentation, such as organic acids, can help extract and solubilise flavonoids contained in the tea (*33*). For these reasons, it is common to observe fluctuations in the total flavonoid concentration during kombucha fermentation, with a decrease and subsequent increase.

Although the final concentration of flavonoids was lower than in other studies, with a total of (51.1±0.1) mg/L, there was a significant increase of 22.5 % at the end of fermentation. In a study carried out by Li *et al.* (*34*), there was a 14 % increase in the flavonoid concentration in the traditional green tea kombucha, increasing from 318.21 to 362.90 mg/L during fermentation. Jakubczyk *et al.* (*35*) obtained a total concentration of (146.8±3.4) mg/L of flavonoids in 7 days of fermentation. According to Gaggìa *et al.* (*31*), the highest concentrations of flavonoids were observed at seven days of fermentation, where they obtained an 11.6 % increase in green tea medium, with a decrease with longer fermentation.

The AA of kombucha is directly related to the concentration of phenolic compounds and their ability to react with free radicals, and this activity increases with the duration of fermentation. A more significant increase in AA can be observed in longer fermentations. For instance, in the study by Chakravorty *et al.* (*6*), the AA determined using DPPH and ABTS methods increased by 39.7 and 38.4 %, respectively, during a 21-day fermentation. However, AA depends on various factors, including the type of substrate, fermentation time and the present microorganisms. Khosravi *et al.* (*36*) reported a 7 % increase in AA determined using DPPH method in a black tea kombucha fermented for 15 days. Chu and Chen (*37*) found a 70 and 40 % increase, respectively, in AA determined using DPPH and ABTS methods in a black tea kombucha fermented for 7 days.

In this study, two antioxidant methods, DPPH and ABTS, were evaluated during seven days of kombucha fermentation. As shown in Table 3, the AA determined by the DPPH method decreased in the first few days, resulting in a decrease of 0.16 % from the first day. However, after the 6th day, the AA started to increase again and was expected to increase further, suggesting that kombucha could be a promising source of antioxidant compounds.

The AA determined by the ABTS method also decreased in the first few days but increased by 18.85 % at the end of the 7th day. These results suggest that kombucha may have a significant antioxidant effect, especially during longer fermentations. One possible explanation for the initial decrease in AA during kombucha fermentation is that, as mentioned earlier, bacteria and yeasts have a higher metabolic activity at the beginning of fermentation and utilise phenolic compounds as substrates for their growth. This could lead to the degradation of these compounds and consequently to a temporary decrease in AA.

However, it is important to note that these initial decreases in AA during fermentation should not be considered negative, as they are a natural and necessary part of the kombucha production process. Moreover, a significant increase in AA can be observed during longer fermentations. AA may also vary depending on the type of test used and other factors, such as the concentration of other bioactive compounds that can affect the AA of kombucha.

#### Kombucha pH and total acidity

Acidification is a fermentative process that uses a symbiotic culture of bacteria and yeast (SCOBY). During fermentation, the bacteria and yeasts present in the SCOBY use the sugar from the tea to produce organic acids, mainly acetic and glucuronic acids, which are responsible for the acidification of the medium (*6*).

The initial pH of the medium was 2.69 and decreased to 2.38 on day 7, indicating that the medium became more acidic during fermentation. The pH sharply decreased between day 2 and 5, and steadily decreased from day 6 to 7. According to Jayabalan *et al.* (*38*) kombucha tends to slow down the decrease in pH after a few days of fermentation due to the buffering effect resulting from the dissociation of carbon dioxide, which occurs through the reaction between the hydrogen ions from the organic acids and the bicarbonate ions.

In addition to the acidification of the medium, an increase in total acidity, expressed in g acetic acid per 100 g, was also observed during fermentation. The production of acetic acid is a characteristic of kombucha production due to the presence of acetic acid-producing bacteria in the SCOBY. The total acidity started at 0.40 g/100 mL and reached 1.09 g/100 mL at the end of day 7. During longer fermentation periods, there can be a significant increase in total acidity, indicating a concentration of organic acids. These acids have antimicrobial activity and an inhibitory effect on acid-intolerant species such as *Escherichia coli*, *Shigella dysenteriae, Salmonella* Typhi and *Vibrio cholera* (*39*).

#### Concentration of RS in kombucha

Reducing sugars are carbohydrates with a free aldehyde or ketone group in their structure, which reduce metal ions such as copper(II) to copper(I) ion in alkaline solutions. Glucose and fructose are examples of reducing monosaccharides. In contrast, sucrose, a disaccharide consisting of one molecule of glucose and one of fructose, does not have a free aldehyde or ketone group and is therefore considered a non-reducing sugar. However, sucrose can become a reducing sugar under certain conditions, such as enzymatic action or acid hydrolysis, which break down the sucrose molecule into its glucose and fructose components, *i.e.* reducing sugars (*40*). This is important for the production of kombucha, as the fermentation of this beverage breaks down the sucrose in the tea into reducing sugars, which are used by the bacteria and yeast in the SCOBY to produce organic acids and other substances.

Table 4 shows the concentrations of reducing sugars throughout the fermentation of kombucha, with their initial concentration in the medium of 24.8 g/L. The breakdown of sucrose into reducing sugars was slower in the first three days and more rapid from day 4 onwards, reaching 77.0 g/L at the end of day 7. This increase in the concentration of reducing sugars is expected in the production of kombucha, since the beverage is produced by fermenting the sucrose contained in the tea.

It is important to note that the final concentration of reducing sugars in the fermentation of kombucha may vary depending on the conditions of the fermentation, such as the amount of sugar added to the tea and the fermentation duration. In this study, 40 % of previously fermented kombucha was used, which means that the used beverage already contained reducing sugars in its composition, and since sucrose is not completely consumed in the first week of fermentation (*8*), this may explain the relatively high concentration of reducing sugars at the end of fermentation.

#### Concentration of TP in kombucha

The digestibility of nutrients contained in the culture medium improved during kombucha fermentation. This benefit is attributed to the SCOBY, which can synthesise enzymes such as proteases and lipases that help break down proteins and lipids contained in the ingredients of the culture medium. As a result, the nutrients are converted into simpler forms, making them more accessible for microbial growth (*41*).

The kinetics of protein concentration in the culture medium was analysed and an increase of 18.6 % was observed in seven days of fermentation, reaching a final value of 0.70 g/L. This result is consistent with the literature, which states that the total protein value should be around 1 g/L (*42*, *43*). It is important to note that the decrease in concentration in the first few days is due to the consumption of nitrogen by the microorganisms during the fermentation process (*36*).

Previous studies, such as Kallel *et al.* (*44*), showed that a total protein value of 0.66 g/L was achieved in fermented green tea medium for six days, corresponding to a growth of 40 %. On the other hand, Sreeramulu *et al.* (*33*) found a protein concentration of approx. 0.20 g/L in 14 days of fermentation with a black tea culture. These differences can be explained by variations in the ingredients of the culture medium, fermentation duration and the strains of microorganisms used in each study.

### WAC of bacterial cellulose

The BC produced in an optimal green tea medium had a water absorption capacity of (524±9) %. This result is consistent with those obtained by other authors when cellulose was produced in a green tea medium. Vieira *et al.* (*45*) obtained (529±4) % and Saibuatong and Phisalaphong (*27*) showed a capacity of 490 % of pure BC. However, the composition of the medium is crucial for the differences found between various studies (*45*). Water absorption is an important property of bacterial cellulose, as it directly affects its application in other fields. In biomedical applications, for example, the ability to retain water is important for maintaining adequate moisture in damaged tissue such as burns or wounds (*46*).

On the other hand, in food applications, water absorption capacity is important to improve the texture and stability of food (*47*). In addition, water absorption can also affect the mechanical properties and porosity, which can be useful in applications such as filtration membranes and support for enzymatic reactions (*48*). Therefore, understanding and controlling the water absorption capacity of bacterial cellulose is crucial for its application in different fields.

### Morphology of the produced BC

**Fig. 2:**
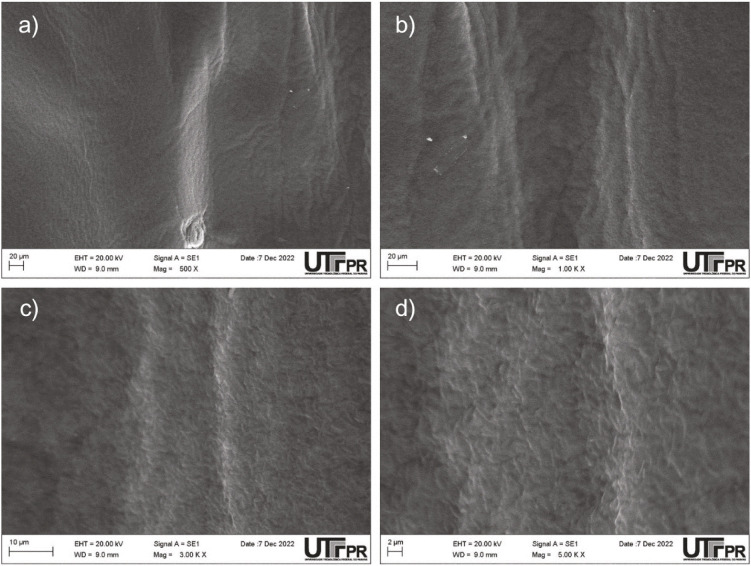
Scanning electron microscopy (SEM) images of the surface of bacterial cellulose (BC) membrane: a) ×500, b) ×1000, c) ×3000 and d) ×5000 magnification

Fig. 2 shows SEM top-view micrographs of the morphology of the BC produced in the optimal medium. The BC had a smooth and uniform surface without large visible sediments. The absence of visible sediments on the surface of the BC indicates a high degree of purity, which is a desirable characteristic for various applications. Additionally, the smooth and uniform surface of the BC is an important factor for its potential use as a scaffold for tissue engineering, as it can provide a suitable environment for cell adhesion and growth. These results demonstrate the effectiveness of using an optimised medium for BC production and highlight the importance of controlling the production parameters to achieve the desired physical and chemical properties of the material.

### Crystallinity of optimised BC

**Fig. 3:**
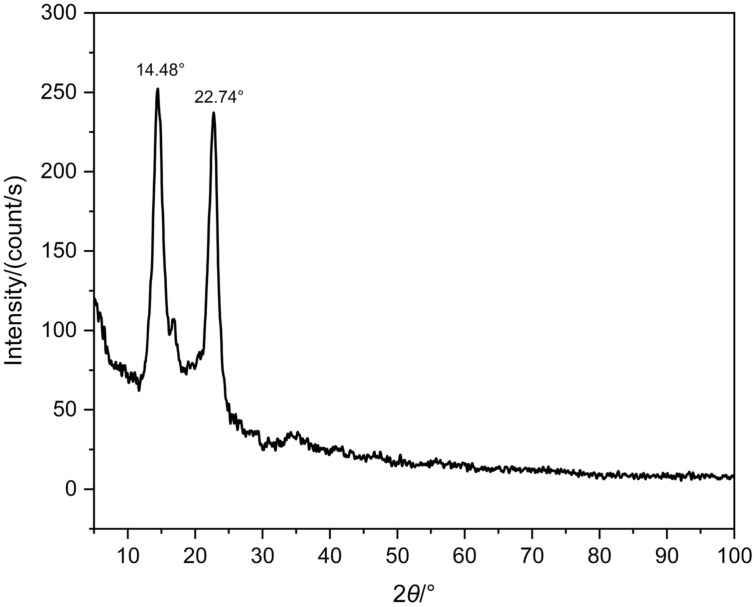
X-ray diffraction (XRD) diffractogram of purified bacterial cellulose (BC)

Fig. 3 shows the X-ray diffractogram of the optimised BC. The peaks observed in the diffractogram correspond to the reflections of the crystalline planes of cellulose, which are indicative of the crystalline structure of the BC. The crystalline structure of BC is composed of long chains organised into fibrils that form crystalline layers intercalated with amorphous regions.

The peaks at 14.48° and 22.74° can be observed for the optimised BC. These peaks were similar to the results shown in the research of Fernandes *et al.* (*49*) for BC produced in green tea with peaks at 14.6° and 22.7°. In addition, the study by Vieira *et al.* (*45*) also showed that the BC membrane in green tea showed two peaks at 15.0° and 22.4°, confirming that these are characteristic peaks of the crystallinity of bacterial cellulose.

The crystallinity index of BC was 79.82 %. This value was higher than that of other studies, *e.g.* Fernandes *et al.* (*49*) obtained a crystallinity index of 53 % for pure BC. Vieira *et al.* (*45*) achieved a crystallinity index of 60 % for BC produced in green tea medium. These results demonstrate the relevance of producing bacterial cellulose in an optimal medium for obtaining a material with high crystallinity, which can have significant implications for future applications.

### Thermal stability of the produced BC

**Fig. 4:**
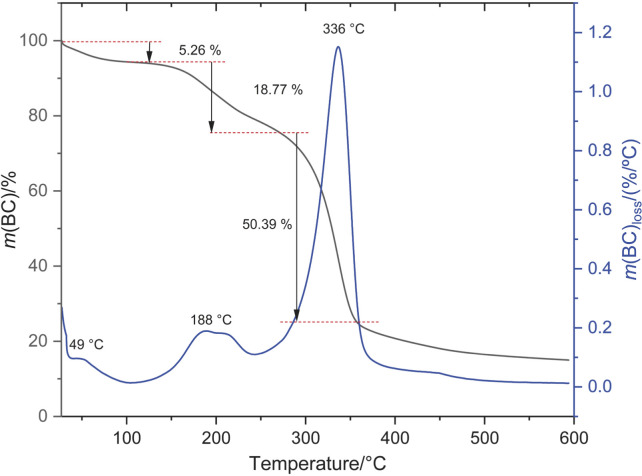
Thermogravimetric analysis (TGA) and derivative thermogravimetric (DTG) of purified bacterial cellulose (BC)

DTG analysis revealed the existence of three distinct decomposition peaks shown in Fig. 4. The first peak, observed at about 49 °C, can be attributed to moisture loss on the membrane surface. The second peak, observed at about 188 °C, can be associated with the decomposition of hemicellulose present in the sample, which is one of the main non-cellulosic components of the bacterial cell wall. The third peak, observed at about 336 °C, can be associated with the decomposition of cellulose itself. This third peak is considered an indicator of the thermal stability of bacterial cellulose, as cellulose decomposition occurs at high temperatures and can be affected by different factors such as the presence of impurities or adsorption of other compounds. The literature suggests that the peaks observed between room temperature and 200 °C are attributed to the presence of water and other substances (*50*), while the peak occurring at temperatures above 300 °C is a result of the decomposition of BC membranes (*51*).

The results of the TGA and DTG analyses show similar behaviour. In the case of TGA, decreases were observed in the TGA curve at three different temperatures: 152, 267 and 359 °C. These decreases represent the decomposition of BC, starting at 152 °C with a loss of 5.26 % of mass, followed by a further decrease up to 267 °C with a loss of 18.77 % of mass, and finally, the third decrease up to 359 °C with a loss of 50.39 % of mass.

The analyses showed that BC has a high thermal stability at elevated temperatures, which could indicate a great potential for its application in the packaging industry. BC can potentially withstand the temperatures used in the sterilisation of packaging, which range from 135 to 150 °C during a high-heat treatment (*52*). However, further studies are needed to evaluate whether the phenolic compounds from the adsorbed BC remain stable after such processes to determine its suitability as active packaging.

## CONCLUSIONS

The results of this study show the successful optimisation of a culture medium for bacterial cellulose (BC) production, as well as the characterisation of the resulting BC membrane and the kinetics of its fermentation. During fermentation, the total phenolic and flavonoid contents of the medium increased significantly and a high antioxidant capacity was observed. These results suggest that kombucha produced with this optimised formulation could be an important source of bioactive compounds for human consumption. Additionally, the BC membrane showed a high water absorption capacity, suggesting that it can be used for the retention of liquids. These results have implications for various sectors, including the food and pharmaceutical industries, and contribute to the development of healthier and more sustainable products. The optimised formulation therefore has excellent potential for the production of a kombucha-like beverage with a high concentration of bioactive compounds as well as for a more productive and efficient production of bacterial cellulose. The BC also has potential applications in packaging due to its high thermal stability during sterilisation and its high adsorption capacity makes it a promising candidate for use in active packaging.

While the study did not provide relevant data showing that coffee grounds can promote the growth of bacterial cellulose, it is worth noting that they have other interesting properties, such as antioxidant and antimicrobial activity. Therefore, it is important not to overlook their potential. However, further research is required to thoroughly investigate their characteristics and properties and evaluate their potential for bacterial cellulose production.
